# Supplementary material for: Content-rich biological network constructed by mining PubMed abstracts
Source: BMC Bioinformatics. 2004 Oct 8;5:147. doi: 10.1186/1471-2105-5-147 (PMC528731; doi:10.1186/1471-2105-5-147)
Supplement: Additional File 2 — The original results of the above study (non-essential files are deleted to keep the file size under the limit set by BMC bioinformatics). [file 1471-2105-5-147-S2.bz2 › chilibotAdditionalFile2/dip05/300ID7486683E1291/html/GRB2_PDGFRB.html]

 


 **GRB2** and **PDGFRB** 
  
Found 14 abstracts in PubMed, retrieved 05.  
 

 What does Google say? 
 PDF only 
| .edu only 

---

**Interactive relationship** (e.g. stimulation, inhibition, etc)

**Neutral relationship**- When  **GRB2** , an adapter protein, was immunoprecipitated from treated VSMCs followed by Western blot analysis with anti phosphotyrosine, PDGFR  [ **PDGFRB** ]  alpha, and  **GRB2**  antibodies, respectively, phosphotyrosine positive staining was observed on PDGFR  [ **PDGFRB** ]  alpha bands of the same blot in stretch stressed VSMCs, supporting the mechanical stress induced activation of PDGFR  [ **PDGFRB** ]  alpha.  Ref: 9737716 FASEB J, 1998

**Non-interactive relationship** (e.g. studied together, co-existance, homology, etc.)

- In conclusion, our results demonstrate that Gi enables the PDGFR  [ **PDGFRB** ]  to signal more efficiently to p42 p44 MAPK, and this appears to be achieved through the regulation of c Src and Grb 2  [ **GRB2** ]  PI3K, which are intermediates in the p42 p44 MAPK cascade.  Ref: 9882612 Biochem J, 1999
